# Parental socioeconomic position and development of overweight in adolescence: longitudinal study of Danish adolescents

**DOI:** 10.1186/1471-2458-10-520

**Published:** 2010-08-29

**Authors:** Camilla Schmidt Morgen, Laust Hvas Mortensen, Mette Rasmussen, Anne-Marie Nybo Andersen, Thorkild IA Sørensen, Pernille Due

**Affiliations:** 1 Institute of Public Health, University of Copenhagen, Denmark; 2 Department of Social Medicine, University of Copenhagen, Copenhagen K, Denmark; 3 National Institute of Public Health, University of Southern Denmark; 4 Institute of Preventive Medicine, Copenhagen University Hospital, Copenhagen, Denmark

## Abstract

**Background:**

An inverse social gradient in overweight among adolescents has been shown in developed countries, but few studies have examined whether weight gain and the development of overweight differs among adolescents from different socioeconomic groups in a longitudinal study. The objective was to identify the possible association between parental socioeconomic position, weight change and the risk of developing overweight among adolescents between the ages 15 to 21.

**Methods:**

Prospective cohort study conducted in Denmark with baseline examination in 1996 and follow-up questionnaire in 2003 with a mean follow-up time of 6.4 years. A sample of 1,656 adolescents participated in both baseline (mean age 14.8) and follow-up (mean age 21.3). Of these, 1,402 had a body mass index (BMI = weight/height^2^kg/m^2^) corresponding to a value below 25 at baseline when adjusted for age and gender according to guidelines from International Obesity Taskforce, and were at risk of developing overweight during the study period. The exposure was parental occupational status. The main outcome measures were change in BMI and development of overweight (from BMI < 25 to BMI > = 25).

**Results:**

Average BMI increased from 21.3 to 22.7 for girls and from 20.6 to 23.6 in boys during follow-up. An inverse social gradient in overweight was seen for girls at baseline and follow-up and for boys at follow-up. In the full population there was a tendency to an inverse social gradient in the overall increase in BMI for girls, but not for boys. A total of 13.4% developed overweight during the follow-up period. Girls of lower parental socioeconomic position had a higher risk of developing overweight (OR's between 4.72; CI 1.31 to 17.04 and 2.03; CI 1.10-3.74) when compared to girls of high parental socioeconomic position. A tendency for an inverse social gradient in the development of overweight for boys was seen, but it did not meet the significance criteria

**Conclusions:**

The levels of overweight and obesity among adolescents are high and continue to rise. Results from this study suggest that the inverse social gradient in overweight becomes steeper for girls and emerges for boys in late adolescence (age span 15 to 21 years). Late adolescence seems to be an important window of opportunity in reducing the social inequality in overweight among Danish adolescents.

## Background

The prevalence of overweight and obesity has increased markedly among children and adolescents in recent years in Denmark as it has internationally[1-7]. Although recent studies suggest that the rapid increase in childhood obesity prevalence may be leveling off[8-10], this does not seems to be the case among adolescents[9]. The teenage years may be important in the life course development of obesity, as obese adolescents often become obese adults[4,11-13] with elevated risk of hypertension, impaired vascular function, type 2 diabetes, systemic inflammation, oxidative stress and coronary heart disease[14,15]. Furthermore overweight and obesity in adolescence may themselves be a more powerful predictors of these risks regardless of overweight and obesity in adulthood[5,16,17]. Overweight adolescents may experience a reduction in the quality of life[18] because of lower self-esteem[19], discrimination[20], poorer body image[19] and poor social and economic outcomes in young adulthood[12,21,22] associated with the early onset of obesity[23,24].

In cross sectional studies, obesity has been shown to be related to socioeconomic position among children and adolescents as suggested by Sobal and Stunkard in 1989[25] and by Shrewsbury and Wardle in 2008[26]. The studies show that in developed countries children from low socioeconomic families appear to be at higher than average risk for overweight and obesity. The opposite gradient is seen in some middle-income countries. In these countries there are also examples of opposite gradients between boys and girls, with an inverse gradient for girls[27]. Ball et al. reviewed the literature on socioeconomic status and weight change among adults in 2005 showing a relatively consistent inverse relationship over time when socioeconomic status was measured as occupation[28]. However, few studies have explored if this is the case for children and adolescents[29-31], and it remains unknown at what age the relationship between socioeconomic position and obesity emerges[25] and whether it leads to a self-promoting vicious cycle in which the psychosocial adverse effects of obesity worsen the obesity[32].

A good understanding of when the inverse social gradient in overweight emerges, if this gradient is changeable and either strengthens or weakens as adolescents move into adulthood may provide useful insights for the development of an effective prevention strategy targeting socioeconomic inequality in overweight. Therefore, we aimed to investigate whether there is an inverse social gradient in overweight and weight change, and whether the inverse social gradient in overweight emerges or changes during late adolescence in a Danish setting.

## Methods

### Sample

The study comprised a sample of 23 municipalities selected to be representative of all municipalities in Denmark, supplemented by a representative sample of schools from the two largest cities in Denmark, Copenhagen and Aarhus. In the municipalities included (except for Aarhus and Copenhagen), the samples comprised all students who underwent the standard health examination by a school physician before leaving school in the school year 1996/97. The health examinations are offered to all schools and take place in both private and public schools.

The storage and linking of the data was approved by the Danish Data Protection Agency and The Danish National Committee on Biomedical Research Ethics approved the collection of data.

**Figure 1:**
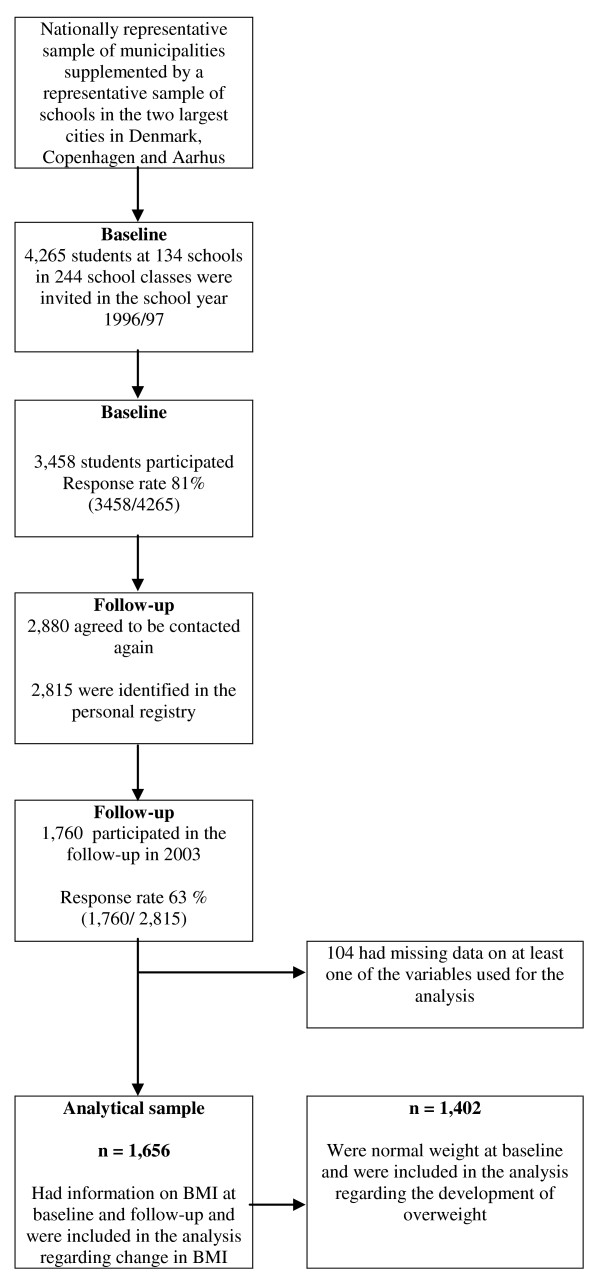
**Flow chart showing the sampling in the study**.

As shown in figure 1, a total of 3,458 14-16-year-old students were enrolled in the cohort at baseline in 1996-97. They were all examined by a school physician, who measured height, weight and reported parents' socioeconomic position. Of those invited, 81 percent participated at baseline. A total of 2,880 agreed to be contacted again.

At follow-up, we identified 2,815 of the 20-22-year-olds in the civil registry by their personal identification number (after permission from the National Board of Health and the Danish Data Protection Agency) and they were invited to participate. The follow-up consisted of a self-administered questionnaire, including self-reported height and weight. A total of 1,760 returned the questionnaire (response rate at 1,760/2,815 = 63%). In the analyses we excluded 104 with missing information on height and weight. Body Mass Index (BMI = weight (kg)/height^2 ^(m)) was calculated for each individual on the basis of measured weight and height at baseline and on the basis of self-reported weight and height at follow-up. Thus, 1,656 participants were eligible for analyses on change in BMI during follow-up, and 1,402 with a normal weight at baseline were eligible for the analysis regarding the development of overweight.

At baseline, girls and individuals with parents from the highest socioeconomic groups were overrepresented when compared to the Danish population in general.

### Exposure

We used socioeconomic position measured by occupation of the parents as exposure. Information about parental socioeconomic position came from the school physician questionnaire and was reported at baseline. The school physician determined parental occupational status from own knowledge about the child, from information from the child and from the teacher. The ranking was based on DISCO-88 classification, which is a Danish version of ISCO-88 used by Statistics Denmark http://www.dst.dk. In order to combine information on paternal and maternal occupational level into one variable we used the highest level of occupational status from either the father or mother, whichever was higher. We worked with eight occupational groups and they were ranked with unemployed as the lowest level and self-employed as the highest level. We used white-collar group 1 as reference, since this group was more homogeneous and bigger than the group of self-employed.

The levels were defined as unemployed, non-skilled manual worker, under education, skilled-manual worker, white-collar group 3, white-collar group 2, white-collar group 1 and self-employed. The categorisation was made from a combination of four questions regarding occupation of the mother and father and a question regarding the reason for not being on the labour market for the mother and the father.

From the questionnaire we have aggregated self-employed with self-employed farmers into one. The group of self-employed included self-employed with and without employees. The unemployed group contains disability retired, housewife (unemployed), unemployed, long time illness (unemployed) and people receiving social security benefit.

### Outcome

There were two primary outcomes: increase in BMI and development of overweight (BMI > = 25, including obese) during the study period.

Change in BMI in the full population was categorised as a continuous measure of upward change in crude BMI "points" (not adjusted for age) from baseline to follow-up.

Since adult BMI criteria for overweight and obesity underestimate the proportion of overweight and obesity among adolescents we categorized the students' weight status using the International Obesity Task Force (IOTF) criteria, which identify BMI for each age in half years differentiated on sex with a predicted BMI of 25 or more at age 18[33]. Students with an age and sex adjusted BMI[33] corresponding to a value below 25 at baseline were categorised as normal weight, and students with a BMI at or above 25 (n = 254) were excluded from the analysis regarding the risk of developing overweight.

Adolescents with an age and sex adjusted BMI corresponding to a value below 25 at baseline and above or at 25 at follow-up were categorized as individuals becoming overweight during the study period.

### Statistical analysis

In this study, students were nested within naturally occurring hierarchies of school physicians (students were examined by 34 different school physicians) and schools (pupils nested in 134 different schools). To account for the cluster sampling of the study, we used multilevel linear regression to examine the change in BMI from baseline to follow-up, adjusting for school physician and school cluster effects and multilevel logistic regression to examine the risk of developing overweight while adjusting for school physician and school cluster effects [34-36]. We did not adjust for school class cluster effects, as there were less than 10 students in all classes in the final analytical sample. We used STATA software version 10.0 and used the procedures XTMELOGIT and XTMIXED.

## Results

### Adolescents lost to follow-up

At baseline a total of 3,458 students were included in the study, as shown in table 1. Of these, 1,802 were excluded from the final analytical sample because they were lost to follow-up or had missing information on key variables. The excluded group was not different from the included group (n = 1,656) regarding BMI and age at baseline, but they had a higher prevalence of parents from lower socioeconomic groups, and a lower proportion of males and of individuals living with both biological parents.

**Table 1:** Descriptive characteristics of the individuals included in the analytical sample (n = 1656) and those excluded because of loss to follow-up or missing information on key variables (n = 1802)

Characterstics		Included (n = 1656)	Excluded (n = 1802)	n in the excluded group*	p-value, χ2/t-test
**Crude mean BMI at** **baseline **(std. dev)		21.0 (3.0)	20.9 (3.1)	1,663	0.53
					
**Age adjusted BMI** **according to IOTF** **criteria**[33]	< 25	84.7%	82.8%	1,087	0.19
	≥25	15.3%	17.2%		
					
**Age at baseline**					
	13	0.6% (10)	0.4% (7)		
	14	24.5% (405)	22.3% (401)		
	15	69.0% (1143)	69.4% (1248)	1,799	0.11
	16	5.7% (95)	7.5% (135)		
	17	0.1% (2)	0.4% (7)		
	18	0.1% (1)	0.1% (1)		
					
**Sex**					
	Male	38.1% (631)	54.5% (982)	1,802	< 0.0001
	Female	61.9% (1025)	45.5% (820)		
					
**Lives with**					
	Biological parents	75.2% (1246)	67.9% (1186)		
	One biological, one cohabitant	11.0% (182)	14.4% (251)	1,748	< 0.0001
	One parent	12.2% (202)	15.2% (265)		
	Other than parents	0.7% (12)	1.6% (28)		
	Not known	0.9% (14)	1.0% (18)		
					
**Socioeconomic** **position**, highest of the parents	Unemployed	5.7% (94)	11.8% (212)		
	Non-skilled manual worker	7.6% (125)	10.2% (184)	1,802	< 0.0001
	Under education	1.3% (21)	2.3% (42)		
	Skilled manual worker	9.5% (157)	10.0% (181)		
	White-collar group 3	19.1% (317)	18.8% (338)		
	White-collar group 2	26.2% (433)	20.9% (377)		
	White-collar group 1	18.7% (309)	13.4% (242)		
	Self-employed	12.1% (200)	12.5% (226)		
					
**Total**		100%	100%		

*Numbers vary according to the number of students with missing information

### Mean BMI, prevalence of overweight and obesity

Table 2 shows the rise in mean crude BMI from 21.3 to 22.7 (mean rise 1.4 (95% CI: 1.3;1.6) p < 0.0001) for girls and from 20.6 to 23.6 (mean rise 3.0 (95% CI:2.8;3.2), p < 0.0001) for boys during the follow-up period. A total of 15.5% (159/1,025) of the girls and 15.1% (95/631) of the boys were overweight (BMI ≥ 25, including obese), when measured at baseline. At follow up the percentages for overweight (obese included) based on self-reported weight and height were 19.5 (200/1,025) and 24.4 (154/631) for girls and boys respectively. A total of 3.0% (31/1,025) of the girls and 1.6% (10/631) of the boys were obese (BMI≥30) at baseline. At follow-up the percentages for obesity were 4.4 (45/1,025) and 4.0 (25/631) for girls and boys respectively.

**Table 2:** Socioeconomic and anthropometric characteristics of the study population by sex (n = 1,656)

Baseline characteristics, 1996-97	Girls, n = 1,025	Boys, n = 631	p-value, χ2/t-test
**Mean age **(SD*)	14.8 (0.5)	14.8 (0.6)	
			
**Average weight **(SD)	58.4 kg (9.7 kg)	62.7 kg (10.8 kg)	< 0.0001
			
**Mean height **(SD)	165.7 cm (6.1 cm)	174.2 cm (7.6 cm)	
			
**Crude mean BMI **(SD) (weight in kg/(height in meters^2^))	21.3 (3.2)	20.6 (2.7)	< 0.0001
			
Age adjusted BMI according to IOTF criteria**			
< 25	84.5% (866)	84.9% (536)	0.16
25 ≤ BMI< 30	12.4% (127)	13.5% (85)	
≥30	3.0% (31)	1.6% (10)	
			
**Socioeconomic position, highest of the household**			
Unemployed	5.9% (60)	5.4% (34)	0.73
Non-skilled manual worker	7.7% (79)	7.3% (46)	
Under education	1.5% (15)	1.0% (6)	
Skilled manual worker	9.9% (101)	8.9% (56)	
White-collar group 3	19.9% (204)	17.9% (113)	
White-collar group 2	26.1% (267)	26.3% (166)	
White-collar group 1	17.7% (181)	20.3% (128)	
Self-employed	11.5% (118)	13.0% (82)	
			
Total	100% 1025	100% 631	

			

**Follow-up characteristics, 2003**	**Girls, n = 1,025**	**Boys, n = 631**	

**Mean age **(SD)	21.2 (0.7)	21.3 (0.7)	
			
**Average weight **(SD)	64.8 kg (11.0 kg)	78.7 kg (11.5 kg)	< 0.0001
			
**Mean height **(SD)	169.0 cm (6.2 cm)	182.6 cm (6.7 cm)	< 0.0001
			
**Crude mean BMI **(SD) (weight in kg/(height in meters^2^))	22.7 (3.6)***	23.6 (3.0)***	< 0.0001
			
**BMI cut points**			
< 25	80.5% (825)	75.6% (477)	0.02
25 ≤ BMI< 30	15.1% (155)	20.4% (129)	
≥30	4.4% (45)	4.0% (25)	
			
Individuals becoming overweight at follow-up**			
Yes, moved from BMI < 25 to BMI ≥25	11.2% (97)	17.0% (91)	0.002
No	88.8% (769)	83.0% (445)	
			
**Individuals becoming normal weight at follow-up**			
Yes, moved from BMI ≥25* to BMI < 25	35.2% (56)	33.7% (32)	0.80
No	64.8% (103)	66.3% (63)	

*Standard deviation

**BMI cut points at baseline according to IOTF criteria[33]

*** Difference from baseline crude mean BMI p < 0.0001.

### Weight development during the six-year study period

As shown in the lower part of table 2, a total of 11.2% (97/866) of the non-overweight girls (BMI corresponding to an age and sex adjusted value below 25) at baseline developed overweight over the six-year study period. 17.0% (91/536) of the non-overweight boys developed overweight during the study period.

A total of 35.2% (56/159) of girls who were overweight at baseline moved to a normal weight over the six years, and 33.7% (32/95) of the overweight boys moved from overweight to normal weight in the study period. The percentages moving to a normal weight range were larger for individuals from families of higher socioeconomic position (data not shown).

### The inverse social gradient in overweight at baseline and follow-up

Results from multilevel logistic regression analysis, shown in table 3, revealed an inverse social gradient in overweight at baseline and at follow-up among girls. Girls from families with an occupational level corresponding to white-collar group 1 had the lowest risk of overweight at baseline and at follow-up. Girls from families with an occupational level corresponding to unemployed, non-skilled manual worker, under education, skilled manual worker, white-collar group 3 to 1 and self-employed had increased risks of overweight, both at baseline and at follow-up when compared to girls from white-collar group 1 families. Though the confidence intervals were wide, the inverse social gradient in overweight seemed steeper at follow-up, indicating that the relative social inequality in overweight was more pronounced among the population of 21-year-old girls than in the population of 15-year-old girls. The two gradients are, however, not calculated in the same number of subjects (1544 versus 1025 included) as we wanted to keep as many individuals as possible in the baseline sample in order to show a social gradient as close as possible to the one in the source population.

**Table 3:** Multilevel logistic regression analysis (OR, 95%CI) of the cross-sectional associations between parental socioeconomic position and overweight at baseline (n = 2743)* and at follow-up (n = 1656)

Socioeconomic position	N	%	% overweight	OR **	95% CI	p-value ***
**Baseline, female**	1544	100				< 0.000
						
Unemployed	107	6.9	13.3	**1.88**	1.11;321	
Non-skilled manual worker	144	9.3	20.3	**3.28**	2.10;5.11	
Under education	29	1.9	20.0	**2.36**	1.07;5.18	
Skilled manual worker	155	10.0	16.8	1.41	0.87;2.28	
White-collar group 3	308	20.0	15.7	**1.95**	1.30;2.90	
White-collar group 2	372	24.1	13.1	**1.51**	1.02;2.24	
White-collar group **1/ref.group**	235	15.2	10.5	1.00		
Self-employed	194	12.6	24.6	**3.63**	2.38;5.51	
						
**Baseline, male**	1199	100				< 0.0001
						
Unemployed	82	6.8	36.5	0.87	0.48;1.57	
Non-skilled manual worker	107	8.9	21.7	0.94	0.57;1.53	
Under education	17	1.4	0.0	0.23	0.05;1.04	
Skilled manual worker	103	8.6	21.4	0.97	0.59;1.60	
White-collar group 3	226	18.9	23.9	1.31	0.89;1.93	
White-collar group 2	295	24.6	6.0	**0.53**	0.35;0.80	
White-collar group 1/**ref.group**	207	17.3	12.5	1.00		
Self-employed	162	13.5	13.4	1.1	0.68;1.62	
						
**Follow-up, female**	1025	100				< 0.000
						
Unemployed	60	5.9	13.3	1.40	0.69;2.86	
Non-skilled manual worker	79	7.7	34.2	5.30	3.11;9.02	
Under education	15	1.5	28.7	**4.75**	1.80;12.54	
Skilled manual worker	101	9.9	26.7	**3.81**	2.30;6.34	
White-collar group 3	204	19.9	19.6	**2.47**	1.56;3.93	
White-collar group 2	267	26.1	19.1	**2.38**	1.52;3.71	
White-collar group 1/**ref.group**	181	17.7	8.8	1.00	-	
Self-employed	118	11.5	21.2	**2.49**	1.49;4.17	
						
**Follow-up, male**	631	100				0.005
						
Unemployed	34	5.4	26.5	1.94	0.78;4.79	
Non-skilled manual worker	46	7.3	34.8	**2.82**	1.28;6.23	
Under education****	6	1.0	0.0	-	-	
Skilled manual worker	56	8.9	32.1	**2.55**	1.22;5.34	
White-collar group 3	113	17.9	35.4	**2.93**	1.58;5.44	
White-collar group 2	166	26.3	19.3	1.28	0.69;2.37	
White-collar group 1/**ref.group**	128	20.3	15.6	1.00		
Self-employed	82	13.0	23.2	1.60	0.78;3.27	

*The number varies from the baseline population at n = 3458 because of missing information on BMI at baseline.

**Adjusted for school physician and school cluster effects.

***P-value for a Likelihood Ratio test of the overall effect of socioeconomic position on overweight.

****No events in this category, excluded from the analysis.

For boys the overall p-value indicated that parental occupational status was associated with overweight at baseline. Based on the OR estimates, however, there seemed to be no clear inverse social gradient in overweight among boys at baseline. At follow-up, based on the OR estimates, an inverse social gradient in overweight was present. Boys from families with a parental occupational level corresponding to non-skilled manual worker, skilled manual worker and white-collar group 3 had a higher risk of overweight when compared to boys from families with a parental occupational level corresponding to white-collar group 1. An inverse social gradient in overweight seemed to emerge in late adolescence for boys. Again, due to attrition, the gradients are measured in two different populations (1199 versus 631 boys included).

Adjusting for school physician and school effects did not alter the results.

### Association between socioeconomic position and change in BMI from baseline to follow-up in the entire study population

Table 4 shows that parental socioeconomic position had an influence on the overall weight change from baseline to follow-up for females, but not for males. The *β *coefficient indicates the change in BMI from baseline to follow-up relative to the reference group, white-collar group 1. Adjusting for school and school physician cluster effects did not alter the results.

**Table 4:** Multilevel linear regression analysis (β-values, 95% CI) of the association between parental socioeconomic position and overall change in BMI from baseline in 1996 to follow-up in 2003 by gender (n = 1656).

Socioeconomic position (highest of the parents)	n	%	Multilevel analysis, two level model β *	p-value**	95% CI
**Female**	1025			0.007	
					
Unemployed	60	9	-0.54		-1.29; 0.21
Under education	79	9	0.55		-0.13; 1.23
Non-skilled manual worker	15	2	0.70		-0.66; 2.05
Skilled manual worker	101	9	**1.04**		**0.42; 1.67**
White-collar group 3	204	19	0.19		-0.33; 0.70
White-collar group 2	267	24	0.21		-0.28; 0.69
White-collar group 1/**ref.group**	181	17	0.00		-
Self-employed	118	13	-0.02		-0.61; 0.58
					
**Male**	631			0.94	
					
Unemployed	150	8	-0.14		-0.10; 0.73
Non-skilled manual worker	168	9	0.39		-0.39; 1.17
Under education	38	2	-0.42		-2.31; 1.47
Skilled manual worker	182	10	0.14		-0.58; 0.87
White-collar group 3	343	19	0.23		-0.36; 0.81
White-collar group 2	412	23	0.10		-0.43; 0.63
White-collar group 1/**ref.group**	270	15	0.00		-
Self-employed	218	12	-0.05		-0.69; 0.59

*Adjusted for school physician and school cluster effects.

**P-value for a Likelihood Ratio test of the overall effect of socioeconomic position on overweight.

### Association between socioeconomic position and the development of overweight

An inverse social gradient was seen in the development of overweight in girls, as shown in table 5. Compared to girls with a parental occupational level corresponding to white-collar group 1, girls in families with an occupational level of white-collar group 2, white-collar group 3, under education and non-skilled manual workers had a significantly higher risk of developing overweight over the six-year study period, between the ages 15 to 21 years. Girls with parents in the unemployed and self-employed groups did not have a higher risk of developing overweight than girls with parents in white-collar group 1.

**Table 5:** Multilevel logistic regression analysis (OR, 95% CI) of the association between parental socioeconomic position and the risk of developing overweight between age 15 and 21 years among non-overweight individuals (n = 1402)

Socioeconomic position (highest of the parents)	n	%	OR	p-value	95% CI
**Female**	866			0.020	
					
Unemployed	52	6.0	1.00		0.36;2.81
Non-skilled man. worker	63	7.3	**4.08**		**2.03;8.22**
Under education	12	1.4	**4.72**		**1.31;17.04**
Skilled manual worker	84	9.7	**3.48**		**1.80;6.75**
White-collar group 3	172	19.9	**2.03**		**1.10;3.74**
White-collar group 2	232	26.8	**2.70**		**1.52;4.79**
White-collar group 1/**ref.group**	162	18.7	1.00		-
Self-employed	89	10.3	1.12		0.51;2.47
					
**Male**	536			0.067	
					
Unemployed	25	4.7	0.60		0.16;2.22
Non-skilled man. worker	36	6.7	1.23		0.54;2.80
Under education	6	1.1	-		-
Skilled manual worker	44	8.2	1.79		0.85;3.78
White-collar group 3	86	16.0	1.87		1.00;3.52
White-collar group 2	156	29.1	1.13		0.63;2.03
White-collar group 1/**ref.group**	112	20.9	1.00		-
Self-employed	71	13.3	1.05		0.53;2.10

*Adjusted for school cluster effects.

**No events in this group, excluded from the analysis.

For boys there was no significant social gradient in the risk of developing overweight in the six-year study period between the mean ages of 15 to 21 years.

Due to the sample size it was not possible to adjust for school physician cluster effects. Adjusting for school cluster effects did not alter the results.

## Discussion

Based on a sample of 1,656 adolescents, we found high rates of overweight and obesity combined at around 15.5% at the age of 15 years and the proportion increased markedly to 19.5% and 24.4% for girls and boys, respectively, from the age of 15 to 21 years. Parental socioeconomic position was associated with the overall rise in BMI from the age of 15 to 21 among girls only. An inverse social gradient was seen in the development of overweight among girls. A tendency for an inverse social gradient in the development of overweight for boys was seen, but it did not meet the significance criteria, possibly due to lack of statistical power because more boys than girls were lost to follow-up. Overweight was associated with parental occupational status for girls at the age of 15 and for both sexes at the age of 21. The gradient emerged for boys and increased for girls during the six years of follow-up.

Adolescence may be a critical period for the development of overweight, as overweight may persist through adult life[4,11,37-39] - resulting in many years of elevated risk of morbidity and mortality.

As seen in previous studies[1,5-7,27], our findings show high rates of overweight in adolescence and the rates increase further into young adulthood.

We found a cross sectional inverse social gradient in overweight at the ages 15 and 21 for girls and at age 21 for boys. The social gradient in overweight among adolescents has been addressed by Sobal and Stunkard in their review from 1989[25], where the authors did not find evidence of a distinct social pattern in obesity in developed countries. The authors did though find a remarkably strong inverse relationship between socioeconomic status and obesity among women[25,40]. In a more recent review by Wardle et al from 2008 the authors concluded that the most prominent pattern was an inverse social gradient in adiposity among adolescents in Western societies[26]. A comparative study on socioeconomic position, macro-economic environment, and overweight among adolescents in 35 countries found that the direction and magnitude of social inequality in adolescent overweight showed large international variation, with inverse social gradients in most Western European countries, but positive social gradients, especially for boys, in some Central European countries[27], supporting the findings of cross-sectional socioeconomic gradients in overweight in this study.

It has not previously been well documented whether weight gain and development of overweight in adolescence is socially patterned or when this pattern emerges. We found that the risk of developing overweight in late adolescence was significantly higher among girls from families with lower socioeconomic positions and that the relative gradient increased for girls in this age span, but this was not statistically significant for boys. The lack of association for boys could be explained by a big loss to follow-up, but as the cross-sectional inverse social gradient is increased for girls, we cannot preclude that the gradient only persists for girls in this age span.

The transition from puberty to adulthood could be particularly challenging for young women, for whom an attractive body image may be of greater importance than for the young men. Being of lower social class origin may make a transition with this expectation more difficult for women than for men, and hence the likelihood of development of obesity may be greater in these women than in men from the same classes[27].

In the review on childhood predictors of adult obesity by Parsons et al from 1999, the authors found no evidence of an association between socioeconomic status in early life and childhood obesity[41]. Though not directly comparable, results from a longitudinal Canadian study provide evidence that effects of neighbourhood disadvantage on children's BMI occur between childhood and early adolescence and in a five-year longitudinal study based on an English population of adolescents Wardle et al. found that the inverse social gradient in overweight was already established at the age of 11 and that no further divergence occurred from the age of 11[29]. These results are not in line with the findings of this study, where the inverse social gradient in overweight changed and got steeper from 15 to 21 years, which may indicate differences between developed countries. Alternatively, it could be explained by the use of different measures of socioeconomic position. Oliver et al. used neighbourhood income and Wardle et al. used residential area as a proxy for the socioeconomic position of the adolescents. Our findings are supported by a recent study by Sherwood et al. based on an American population of adolescents in which girls from families of lower socioeconomic position were at increased risk of developing overweight. No association was seen among boys. Socioeconomic position was primarily measured by parental education and, in addition, occupation and eligibility for public assistance[30].

### Strengths and limitations

This study is based on a nationally representative sample of adolescents, which increases the external validity. Information regarding height and weight at baseline was measured objectively, while at follow-up they were self reported. The main weakness of the study is the large attrition, which may bias the results. Participants who did not attend follow-up, were more often from families with lower socioeconomic position, and were less often living with both biological parents, all factors contributing to the risk of selection bias. Since more boys than girls were lost to follow-up, we might have underestimated the social inequality in the development of overweight among boys. These results should therefore be interpreted with caution. However, as the group lost to follow-up did not differ with regards to BMI at baseline, and since the participation rate in the school physician examinations (at baseline) is generally high, we believe that the associations found are not severely biased due to loss to follow-up.

Another limitation of the study is the self-reported measurements of height and weight at follow-up, since self-reporting tends to lead to an underreporting of BMI at follow-up[42,43]. This potential misclassification might lead to an underestimation of the number of participants who develop overweight and an overestimation of the number of participants who achieve a healthy weight range during the six-year study period. Two previous studies have found misclassification to be larger among adolescents from lower socioeconomic groups[44,45], which would mean that associations in our study are underestimated. A false association between parental occupational status and the longitudinal development of overweight would appear if participants from families with a lower socioeconomic position at baseline were more likely to under- or over report weight at follow-up. We have not been able to find research covering this subject.

The use of BMI as a measure of weight status has been criticised because it is not sex-specific for adults and may be confounded by skeletal structure[46]. More precise measures such as waist circumference and DEXA scans were not available and BMI was suitable at follow-up to secure the least degree of non-response. BMI is though a widely used and accepted measure in epidemiological studies.

The use of parental occupational status as the exposure had limitations as the information from the questionnaire was difficult to aggregate into fewer socioeconomic groups. A further aggregation of the categories would have made the interpretation unclear and would have thrown away too much of the information given in the questionnaires.

A further limitation of the exposure used is the fact that the source of information was the school physician, who determined parental occupational status from its own knowledge about the child, from information from the child and from the school teacher. How thorough school physicians were in obtaining this information may have differed. We have, though, considered this possible bias to be non-differential as we find it most likely that the proportion of misclassified individuals does not depend on the later risk of obesity.

Parental occupational status is one of many ways to quantify the socioeconomic position of adolescents and it seems to have an influence on weight gain and risk of obesity that is independent of the parents' own degree of obesity[47]. Information on parental educational and income levels might improve the understanding of the factors and processes that create the socioeconomic disparities in overweight and the development of overweight among adolescents.

## Conclusions

The levels of overweight and obesity among adolescents are high and continue to rise. In our study, the results suggest that the inverse social gradient in overweight gets steeper for girls and arises for boys in late adolescence. Late adolescence seems to be an important window of opportunity in reducing the social inequality in overweight among Danish adolescents.

## Competing interests

The authors declare that they have no competing interests.

## Authors' contributions

The CSM, ANA and PDU author designed and initiated the study. CSM and LHM author performed the statistical analyses. All authors helped gather or interpret data and write the article. All authors approved the final version of the article. The study complies with the Helsinki declaration on ethics in science. There are no conflicting interests regarding the funding of the study. The storage and linking of the data were approved by the Danish Data Protection Agency, and The Danish National Committee on Biomedical Research Ethics approved the collection of data.

## Pre-publication history

The pre-publication history for this paper can be accessed here:

http://www.biomedcentral.com/1471-2458/10/520/prepub
